# The perks of doing housework: Longitudinal associations with survival and underlying mechanisms

**DOI:** 10.1186/s12877-023-04039-1

**Published:** 2023-06-06

**Authors:** Li Chu, Xianmin Gong, Jennifer C. Lay, Fan Zhang, Helene H. Fung, Timothy Kwok

**Affiliations:** 1 Department of Psychology, Stanford University, Stanford, USA; 2 Department of Psychology, The Chinese University of Hong Kong, Hong Kong SAR, China; 3 Big Data Decision Analytics Research Centre, The Chinese University of Hong Kong, Hong Kong SAR, China; 4 Department of Psychology, University of Exeter, Exeter, England; 5 Department of Public Health and Preventive Medicine, School of Medicine, Jinan University, Guangzhou, China; 6 Department of Medicine and Therapeutic, The Chinese University of Hong Kong, Hong Kong SAR, China

**Keywords:** Healthy aging, Housekeeping, Cognitive function, Mental health, Exercise

## Abstract

**Background:**

Although the majority of existing literature has suggested positive effects of housework on older adults’ health and survival rate, the underlying mechanisms of such effects remain unclear. To address potential mechanisms, the present study examined the association between older adults’ housework engagement and days of survival across 14 years and tested three potential mediation pathways in this association.

**Methods:**

Four thousand Hong Kong older adults (50% female; aged between 65 and 98 years) participated in a longitudinal study in which they reported initial housework engagement and health status across three domains (cognitive functioning, physical health, and mental health) at the baseline, and the numbers of days they survived over the subsequent 14-year period were recorded. Linear regression, Cox proportional hazard, and parallel mediation analyses were performed to examine the relationship between housework engagement and days survived, and the mediating effects of these three health factors.

**Results:**

The results showed a positive association between housework engagement and days survived after controlling for demographic variables (age, sex, education, marital status, subjective social status, and living alone). Physical health and mental health, but not cognitive functioning, partially mediated the relationship between housework engagement and days survived. The findings suggest that doing housework may contribute to longer survival by improving older adults’ physical and mental health.

**Conclusion:**

The current study confirms positive relations of housework with health and mortality among Hong Kong older adults. As the first study examining the relationships and mediation pathways between doing housework and survival in later life, the findings advance our understanding of the mechanisms underlying the positive association between housework and mortality and provide insights for future daily-life health-promotion interventions for older adults.

**Supplementary Information:**

The online version contains supplementary material available at 10.1186/s12877-023-04039-1.

## Introduction

Previous research suggests that older adults who spend more time doing housework may have better health and reduced mortality risk [1–3]. Given that post-retirement life is usually more home-based, it is crucial to understand how and why doing housework may be beneficial for older adults. In particular, during the COVID-19 pandemic, as social distancing policies have restricted older adults’ physical activities and social engagement [4], home-based physical activities have become a major source of exercise for maintaining health and well-being [5]. The present study aimed to examine the association between housework and survival, and potential mediating roles of physical health, cognitive functioning, and mental health, using data on mortality risk over a 14-year period among adults aged 65 years and above.

Housework is an important part of everyday life, and potentially even more so in later life. After retirement, the majority of older adults spend most of their waking hours at home, and activity engagement shows a shift away from community-based activities and towards more home-based and family-related activities [6]. In a survey of customary physical activity among 1,042 older adults, 86% of the participants performed indoor activities, and 95% of indoor activity time was spent on housework [7]. Such high levels of engagement in housework may have mixed effects on health and well-being. On the one hand, previous research has revealed how the housework-related “burden” may lead to increased risk of physical symptoms and poor self-reported health [8–11]. On the other hand, housework may be an important source of physical activity [2, 12, 13] and cognitive stimulation in later adulthood [14], with effects comparable in size to those of moderate-to-vigorous physical exercise (e.g., weight training) in enhancing physical and mental health [15]. If housework also involves caregiving (e.g., cooking for family members [16]), it may further enhance social bonds with close others. Such “hidden benefits” of doing housework may counteract the negative impacts of housework-related “burden”, particularly among older adults who may rely more on in-home activities as a way of maintaining well-being. In the following sections, we discuss these three main benefits of housework in detail.

Doing housework may have beneficial effects on physical health that are similar to those of physical activity. Spending more hours on housework has been linked with having higher levels of physical activity [2, 13, 17]. Previous research has shown that for people doing full-time sedentary jobs, housework and caregiving may compensate for a lack of physical activity, and increase the likelihood of meeting recommended physical activity levels (i.e., at least 150 min of moderate-to-vigorous physical activity per week [18, 19]). Indeed, the effect of housework on mortality risk is similar to that of total physical activity. Chen et al. found that, compared to people with higher household activity levels, people with lower household activity levels showed a 60% increase in all-cause mortality risk [12].

Doing housework may also benefit older adults’ cognitive functioning, which in turn is associated with better health and longer life expectancy. It has been found that engaging in housework was associated with better general cognitive functioning and recall ability, particularly among older adults over the age of 80 [20]). Neuroimaging research has offered more direct evidence, specifically that household physical activity (e.g., light housework and caregiving), but not recreational physical activity, was positively associated with gray matter volume, particularly in the regions of the hippocampus and frontal lobe [21]. Therefore, it is plausible that housework may help delay the onset or progress of cognitive decline.

Beneficial effects of household activities have also been found for mental health. Household physical activities, such as gardening and heavy housework, seem to benefit older adults’ mental health, particularly in reducing fatigue [22]. A recent study showed that greater engagement in household physical activity was associated with fewer depressive symptoms during the COVID-19 pandemic [5]. In addition to mental health benefits stemming from its physical activity component, housework may also enable a person to meet familial obligations, thereby fostering role fulfillment and familial social support [23].

Moreover, potential sex differences in the effects of housework should be taken into account. The beneficial effects of housework have been found more consistently among men than among women [2, 8, 9, 11]. For example, older men who engaged more in heavy and light housework showed a reduced risk of cancer mortality, but this link was not significant among older women [3]. It is possible that older men might be happier and less stressed when doing housework, which contributes to better well-being and health [24]. Nevertheless, sex differences in the effects of housework on mortality need closer investigation. Therefore, we also examine the moderating role of sex in the proposed mediation model linking housework with reduced mortality.

In summary, existing research suggests that housework may improve older adults’ physical health, cognitive functioning, mental health, and eventually, increase life expectancy. However, to our best knowledge, little research has been conducted to address the effects of housework via the three pathways mentioned above. Hence, we propose a more comprehensive model to better understand how doing housework affects mortality. By analyzing a large-scale longitudinal dataset (Mr. & Ms. Os Hong Kong cohort study [25, 26]), the current study has two research aims: (1) to examine the association between housework and mortality (measured as days survived) among older Hong Kong adults; and (2) to examine the potential mediating roles of physical health, mental health, and cognitive functioning in this relationship. We hypothesized that doing more housework would predict more days survived, via promoting older adults’ physical health, cognitive functioning, and mental health.

## Methods

### Data and sample

The data used in the current study were from the 14-year Mr. & Ms. Os Hong Kong cohort study [25, 26]. In our analyses, we focused on the baseline assessment, which was conducted between August 2001 and December 2003. We also used survival information collected in the final wave between November 2015 and September 2017 (i.e., a 14-year follow-up). In the current analyses, we were particularly interested in the relationship between housework (assessed at the baseline) and mortality (number of days survived since the baseline assessment), as well as the potential roles of physical health, cognitive functioning, and mental health (assessed at the baseline) in this relationship.

A total of 2,000 Chinese men (*M*_age_ = 72.39 years, *SD* = 5.00; range 65–92 years) and 2,000 women (*M*_age_ = 72.58 years, *SD* = 5.34; range 65–98 years) in Hong Kong were recruited via advertisements to participate in the baseline assessment (see [26] for more information). The current study was reviewed and approved by the Research Ethics Committee, Chinese University of Hong Kong, and all participants were asked to provide written consent before taking part in the study. At the final wave, a total of 1,658 participants were deceased (41.45%). For detailed sample information, see Table 1.

**Table 1:** Sociodemographic Characteristics of Participants (N = 4,000)

Variables	Descriptive statistics
Age (years)	72.49 ± 5.18, range [65, 98]
Sex	
Male	2,000 (50.00%)
Female	2,000 (50.00%)
Education	
Some primary school	2,137 (53.43%)
Primary school (graduated)	533 (13.33%)
Some secondary school	480 (12.00%)
Secondary school (graduated)	434 (10.85%)
Some postsecondary education	75 (1.88%)
Postsecondary education (graduated)	333 (8.33%)
Some graduate education	0
Graduate school (graduated)	8 (0.20%)
Subjective social status	4.55 ± 1.90, range [1, 10]
Marital status	
Married or in marriage-like relationship	2,829 (70.73%)
Other (e.g., single, divorced, widowed)	1,171 (29.28%)
Living status	
Alone With others	546 (13.65%) 3,454 (86.35%)
Housework	1.69 ± .94, range [0, 6]
Days survived	4933.34 ± 1519.45 days, range [29, 6378] (~ 13.52 ± 4.16 years, range [.08, 17.47])
Cognitive functioning #	30.19 ± 2.03, range [16.35, 33.45]
Physical health #	48.56 ± 8.43, range [11.54, 67.19]
Mental health #	55.44 ± 7.29, range [14.38, 69.53]

*Notes.* Data are presented as *n* (%) or mean ± standard deviation

# Higher scores indicate better functioning/health

### Measures

#### Days survived

We used the number of days survived since the baseline assessment as a measure of mortality (*M* = 4,933, *SD* = 1519.45, range = 29 − 6,378).

#### Housework

At the baseline assessment, participants indicated whether they had engaged in each of six types of housework in the past seven days: (1) light indoor housework (e.g., cleaning, washing dishes, washing/ironing/drying clothes, cooking, buying meals), (2) heavy indoor housework (e.g., vacuuming, cleaning the floor, washing windows, washing vehicles, moving furniture, moving petroleum gas can), (3) home repair work, (4) lawn or yard work, (5) outdoor gardening, and (6) caregiving. Each item was coded as 0 (no) and 1 (yes), indicating whether the individual had engaged in that type of housework. A sum score of the six types of housework was then used as a measure of overall engagement in housework, with higher scores indicating higher levels of housework engagement. The survey items were drawn from the Physical Activity Scale for Elderly (PASE; [27]). The original scale weights each item in order to capture the physical demands of each activity. However, we did not weight the housework items in this study because our focus was housework engagement rather than physical activity levels. By not applying the weighting, we aimed to reduce the conceptual overlap between housework (our main independent variable) and physical health (one of the mediators in our model). Moreover, additional analyses revealed that applying the weights to the housework items did not alter the pattern of results reported below (see Supplementary Table S1 for details).

#### Cognitive functioning

Cognitive functioning was assessed at the baseline using the Chinese version of the Community Screening Interview for Dementia (CSI-D; [28]). The CSI-D is a widely validated tool assessing general cognitive functioning, whose Chinese version has also been validated [29], and it uses interview questions spoken aloud. Higher CSI-D scores indicate higher levels of cognitive functioning. Scores equal to or above 29.5 indicate normal cognitive function, scores below 28.4 indicate possible dementia, and scores between 28.4 and 29.4 indicate a critical state in between normal cognitive functioning and dementia.

#### Physical health & mental health

Physical health and mental health were assessed at the baseline using the 12-Item Short Form Health Survey (SF-12). SF-12 has been frequently utilized to assess physical and mental health, which may indicate people’s quality of life, and it includes questions asking about physical and emotional problems over the past four weeks. Specific formulas were applied to scores on these items to generate two summary scores: the physical component summary score (PCS-12) and the mental component summary score (MCS-12; [30]). These scores were used to index physical health and mental health, respectively, with higher scores indicating better physical/mental health. The two scales have been validated and have shown good reliability in Hong Kong adult samples [31].

#### Covariates

Certain demographic characteristics measured at the baseline were included as covariates: age, sex (0 = male, 1 = female), education (1 = some primary school, 2 = primary school (graduated), 3 = some secondary school, 4 = secondary school (graduated), 5 = some postsecondary education, 6 = postsecondary education (graduated), 7 = took some graduate courses, 8 = graduate school (graduated)), marital status (0 = not married, 1 = married or living in a marriage-like relationship), living status (0 = living with others, 1 = living alone), and subjective social status. Subjective social status was measured using the MacArthur Scale [32]. The scale consists of a 10-rung ladder, with people of the highest social class in Hong Kong being at the top of the ladder and those of the lowest social class at the bottom. Participants were asked to think about their own social status and select a rung on the ladder to represent their positions in the Hong Kong population. We performed multiple imputation for participants with partial missing data using the *mice* package [33] in R [34].

### Analytical strategy

**Fig. 1:**
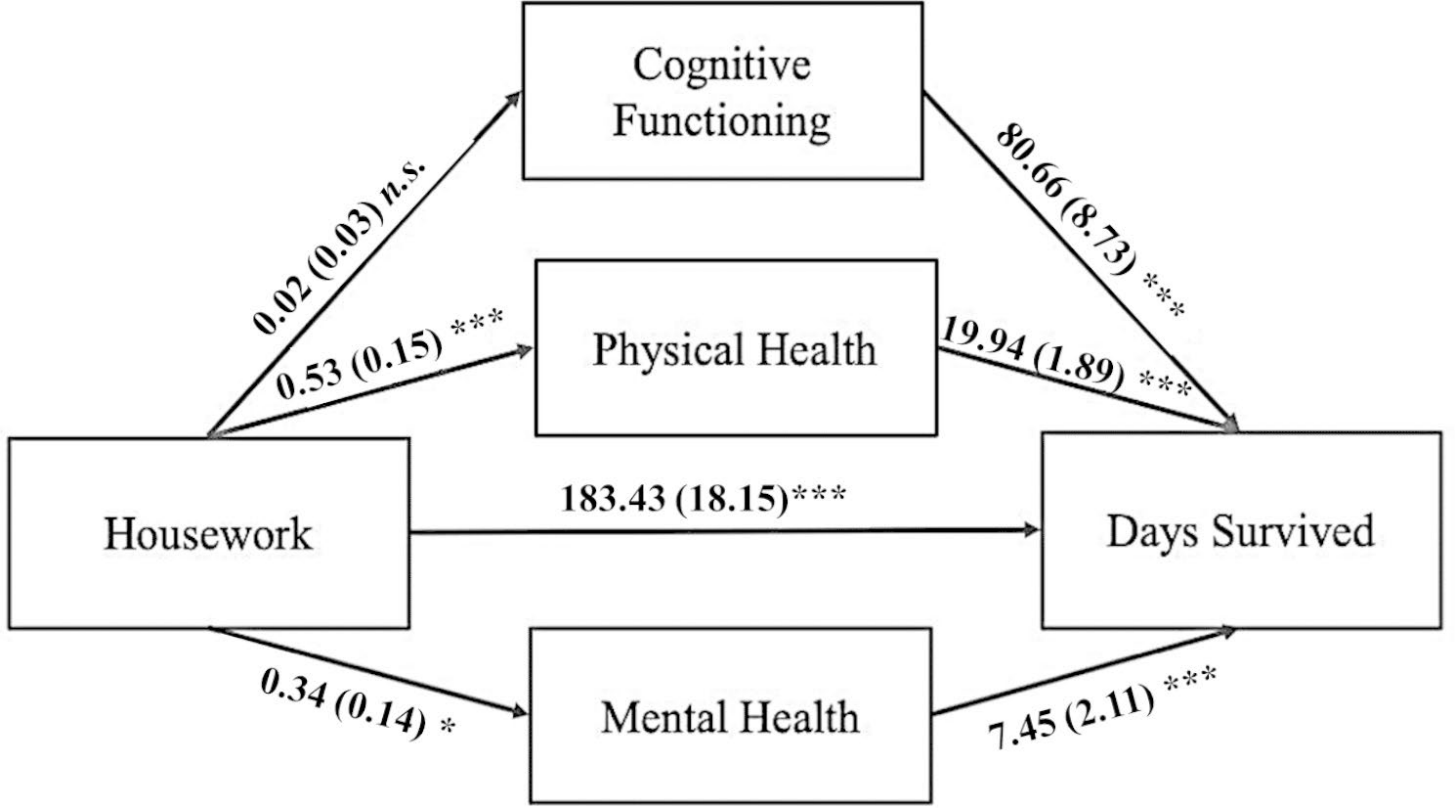
Results of the Multiple Parallel Mediation Model *Notes*. The effect of housework on survival is partially mediated by physical health and mental health, but not by cognitive functioning. This analysis controlled for age, sex, education, living status, marital status, and subjective social status for both the mediators and the outcome variable. Indicated values represent each path coefficient and standard error. *p < .05, **p < .01, ***p < .001, *n.s.* = not significant

As shown in Fig. 1, we examined whether baseline cognitive functioning, physical health, and mental health mediated the relationship between baseline housework engagement and number of days survived (over the 14 years after the baseline assessment) in our sample.

First, we examined the correlations among these variables (as shown in Table 2). Second, we examined the main effect of housework on survival using linear regression and Cox proportional hazard analysis. Third, we examined a parallel mediation model that included all three mediator variables. Demographic variables (age, sex, education, marital status, subjective social status, and living alone) were included as covariates in these analyses (except for the correlation analyses). Note that the survival data were right censored: Some participants were still alive after the study ended, and their days of survival are therefore unknown. To deal with the censoring issue, the lava package in R [34, 35] was used to perform the linear regression and mediation analysis, and the survival package [36] in R was used to perform the Cox proportional hazard analysis.

**Table 2:** Correlations (and Confidence Intervals) Among Variables

Variable	1	2	3	4	5	6	7	8	9	10
1. Housework	-									
2. Days survived	0.15*** [0.12, 0.18]	-								
3. Cognitive functioning	0.04* [0.01, 0.07]	0.11*** [0.08, 0.14]	-							
4. Physical health	0.05*** [0.02, 0.08]	0.08*** [0.05, 0.11]	0.18*** [0.15, 0.21]	-						
5. Mental health	0.01 [-0.02, 0.05]	0.01 [-0.02, 0.04]	0.06*** [0.03, 0.09]	0.02 [-0.01, 0.05]	-					
6. Age	− 0.19*** [-0.22, − 0.16]	− 0.32*** [-0.35, − 0.29]	− 0.26*** [-0.29, − 0.23]	− 0.06*** [-0.09, − 0.03]	0.03* [0.00, 0.06]	-				
7. Sex (0 = male; 1 = female)	0.05** [0.02, 0.08]	0.12*** [0.09, 0.15]	− 0.34*** [-0.37, − 0.32]	− 0.23*** [-0.26, − 0.20]	− 0.05*** [-0.08, − 0.02]	0.02 [-0.01, 0.05]	-			
8. Marital status (0 = not married; 1 = married)	0.05** [0.02, 0.08]	− 0.09*** [-0.12, − 0.06]	− 0.17*** [-0.20, − 0.14]	− 0.06*** [-0.09, − 0.02]	− 0.03 [-0.06, 0.00]	0.20*** [0.17, 0.23]	0.23*** [0.20, 0.26]	-		
9. Education	− 0.03 [-0.06, 0.01]	0.07*** [0.03, 0.10]	0.38*** [0.36, 0.41]	0.13*** [0.10, 0.16]	0.07*** [0.04, 0.10]	− 0.13*** [-0.16, − 0.10]	− 0.27*** [-0.30, − 0.24]	− 0.11** [-0.14, − 0.08]	-	
10. Living status (0 = with others; 1 = alone)	− 0.02 [-0.05, 0.01]	0.06*** [0.03, 0.09]	0.13*** [0.10, 0.16]	0.03 [-0.00, 0.06]	0.04** [0.01, 0.07]	− 0.17*** [-0.20, − 0.14]	− 0.19*** [-0.22, − 0.16]	− 0.50** [-0.52, − 0.48]	0.07*** [0.04, 0.10]	-
11. Subjective social status	− 0.05** [-0.08, − 0.02]	0.03 [-0.01, 0.05]	0.02 [-0.02, 0.05]	0.12*** [0.09, 0.15]	0.09*** [0.06, 0.12]	− 0.01 [-0.04, 0.02]	0.05** [0.02, 0.08]	− 0.05** [-0.08, − 0.02]	0.22*** [0.19, 0.25]	0.04* [0.01, 0.07]

*Notes.* Values in square brackets indicate the 95% confidence intervals of the correlation coefficients. *p < .05, **p < .01, *** *p* < .001

## Results

### Correlations

We first examined the bivariate correlations among our key variables and covariates. We found a weak but positive correlation between housework and number of days survived (*r* = .15, *p* < .001). Housework was also weakly but positively correlated with physical health (*r* = .04, *p* < .01) and cognitive functioning (*r* = .04, *p* = .01), but not mental health (*r* = .01, *p* = .35). Although the effect sizes are relatively small, the results generally support the proposed associations among the variables, qualifying the follow-up Cox proportional hazard analysis and mediation analysis. All covariates showed significant correlations with at least one of the key variables, so we retained all of them in the main analyses. The correlational results are summarized in Table 2.

### Linear regression and cox proportional hazard analysis

Next, we examined the main effect of housework on the number of days survived. The linear regression results showed a significant positive association regardless of whether models controlled for demographic covariates (before controlling for covariates: unstandardized *b* = 531.57, standardized *β* = 0.15, *SE* = 62.82, *p* < .001; after controlling for covariates: unstandardized *b* = 280.46, standardized *β* = 0.08, *SE* = 58.74, *p* < .001). This suggests that people who did more types of housework tended to survive longer. To confirm the association, we performed a Cox proportional hazard analysis and found a consistent result, regardless of whether models controlled for demographic covariates (before controlling for covariates: *HR* = 0.79, *p* < .001, 95% *CI* = [0.74, 0.83]; after controlling for covariates: *HR* = 0.87, *p* < .001, 95% *CI* = [0.82, 0.92]). Based on the hazard ratio (controlling for demographic covariates), there was an approximately 13% reduction in mortality risk with every one additional type of housework engaged in. The Cox proportional hazard results are summarized in Table 3.

**Table 3:** Cox Proportional Hazard Modeling Time to Death Among Older Adults in Hong Kong

Variables	Original Model *HR* [95% *CI*]	Model Without Covariates *HR* [95% *CI*]
Housework	0.87*** [0.82, 0.92]	0.79*** [0.74, 0.83]
Age	1.10*** [1.09, 1.11]	
Sex (0 = male; 1 = female)	0.46*** [0.41, 0.51]	
Marital status (0 = not married; 1 = married)	0.83* [0.72, 0.96]	
Education	0.92*** [0.89, 0.95]	
Living status (0 = with others; 1 = alone)	0.93, *n.s.* [0.79, 1.09]	
Subjective social status	1.00, *n.s.* [0.97, 1.02]	

*Notes*. *n.s. p* > .05, * *p* < .05, *** *p* < .001

We also tested the moderating role of sex on the association between housework and survival using both linear regression and Cox proportional hazard analysis. The results showed a significant sex × housework interaction in both the linear regression model (unstandardized *b* = -316.56, standardized *β* = -0.05, *SE* = 127.71, *p* = .013) and the Cox model (*HR* = 1.15, *p* = .030, 95% *CI* = [1.01, 1.31]), after controlling for demographic covariates. The results suggest that the effect of housework on survival was weaker for women compared to men.

### Mediation analysis

After confirming the main effect of housework on survival, we performed a multiple parallel mediation analysis to examine potential underlying mechanisms. We found a significant partial mediation of the housework-survival days association through the indirect effects of physical health (unstandardized *b* = 10.61, *SE* = 3.21, *p* < .001, 95% *CI* = [4.32, 16.89]) and mental health (unstandardized *b* = 2.56, *SE* = 1.25, *p* = .040, 95% *CI* = [0.12, 5.00]), but not cognitive functioning (unstandardized *b* = 1.23, *SE* = 2.64, *p* = .642, 95% *CI* = [-3.95, 6.40]). The total effect of housework on survival in this parallel mediation model was significant (unstandardized *b* = 197.82, *SE* = 18.57, *p* < .001, 95% *CI* = [161.42, 234.23]), and the detailed results of this model are presented in Fig. 1; Table 4 (the model titled “Original Model”).

**Table 4:** Path Coefficients for the Multiple Parallel Mediation Models (N = 4,000)

	Original Model					Model with Sex Moderation			
	*b*	*β*	*p*	95% *CI*		*b*	*β*	*p*	95% *CI*
**Regressions**									
housework→ survival	183.43	0.13	< 0.001	[147.86, 219.00]		254.50	0.17	< 0.001	[211.88, 297.12]
housework → cognitive	0.02	0.01	0.642	[-0.05, 0.08]		0.03	0.01	0.424	[-0.05, 0.11]
housework → physical	0.53	0.05	< 0.001	[0.23, 0.83]		0.51	0.05	0.006	[0.14, 0.87]
housework → mental	0.34	0.04	0.012	[0.08, 0.61]		0.29	0.03	0.077	[-0.03, 0.61]
cognitive → survival	80.66	0.14	< 0.001	[63.54, 97.77]		214.06	0.34	< 0.001	[182.93, 245.19]
physical → survival	19.94	0.14	< 0.001	[16.24, 23.64]		22.30	0.15	< 0.001	[16.9, 27.7]
mental → survival	7.45	0.05	< 0.001	[3.31, 11.59]		11.90	0.07	< 0.001	[5.84, 17.96]
*(Regressions on covariates)*									
age → survival	-104.57	− 0.45	< 0.001	[-110.85, -98.29]		-112.53	− 0.46	< 0.001	[-118.84, -106.23]
sex → survival	978.75	0.41	< 0.001	[904.85, 1052.65]		490.41	0.19	< 0.001	[418.05, 562.78]
education → survival	54.00	0.07	< 0.001	[32.5, 75.49]		12.48	0.02	0.251	[-8.82, 33.78]
marital → survival	233.94	0.09	< 0.001	[145.53, 322.36]		16.09	0.01	0.720	[-71.94, 104.13]
living status → survival	117.56	0.03	0.028	[12.7, 222.42]		46.23	0.01	0.388	[-58.79, 151.25]
social status → survival	3.20	0.01	0.690	[-12.55, 18.95]		-28.63	− 0.05	< 0.001	[-44.28, -12.97]
age → cognitive	-0.08	− 0.19	< 0.001	[-0.09, -0.06]		-0.08	− 0.20	< 0.001	[-0.09, -0.07]
sex → cognitive	-0.91	− 0.22	< 0.001	[-1.03, -0.79]		-0.91	− 0.22	< 0.001	[-1.03, -0.79]
education → cognitive	0.38	0.30	< 0.001	[0.34, 0.41]		0.38	0.30	< 0.001	[0.34, 0.41]
marital → cognitive	0.32	0.07	< 0.001	[0.17, 0.48]		0.33	0.07	< 0.001	[0.17, 0.49]
living status → cognitive	-0.05	− 0.01	0.624	[-0.24, 0.14]		-0.05	− 0.01	0.631	[-0.24, 0.14]
social status → cognitive	-0.10	− 0.10	< 0.001	[-0.13, -0.07]		-0.10	− 0.10	< 0.001	[-0.13, -0.07]
age → physical	-0.08	− 0.05	0.002	[-0.14, -0.03]		-0.09	− 0.06	0.001	[-0.14, -0.04]
sex → physical	-4.10	− 0.24	< 0.001	[-4.67, -3.53]		-4.09	− 0.24	< 0.001	[-4.66, -3.53]
education → physical	0.24	0.05	0.005	[0.07, 0.4]		0.24	0.05	0.005	[0.07, 0.41]
marital → physical	-0.38	− 0.02	0.307	[-1.11, 0.35]		-0.39	− 0.02	0.300	[-1.12, 0.34]
living status → physical	-0.34	− 0.01	0.455	[-1.22, 0.55]		-0.35	− 0.01	0.440	[-1.24, 0.54]
social status → physical	0.39	0.09	< 0.001	[0.26, 0.52]		0.41	0.10	< 0.001	[0.28, 0.54]
age → mental	0.07	0.05	0.002	[0.03, 0.12]		0.07	0.05	0.002	[0.03, 0.12]
sex → mental	-0.62	− 0.04	0.016	[-1.13, -0.12]		-0.62	− 0.04	0.015	[-1.13, -0.12]
education → mental	0.20	0.04	0.008	[0.05, 0.35]		0.19	0.04	0.011	[0.04, 0.34]
marital → mental	0.13	0.01	0.702	[-0.52, 0.78]		0.12	0.01	0.713	[-0.53, 0.77]
living status → mental	0.83	0.04	0.038	[0.05, 1.62]		0.85	0.04	0.033	[0.07, 1.64]
social status → mental	0.29	0.08	< 0.001	[0.18, 0.41]		0.33	0.09	< 0.001	[0.21, 0.44]
*(Moderating effects of sex)*									
housework × sex → survival						-25.60	− 0.01	0.510	[-101.76, 50.56]
housework × sex → cognitive						-0.05	− 0.01	0.473	[-0.18, 0.09]
housework × sex → physical						0.04	0.002	0.896	[-0.59, 0.67]
housework × sex → mental						0.10	0.01	0.712	[-0.45, 0.66]
cognitive × sex → survival						-210.19	− 0.28	< 0.001	[-245.53, -174.85]
physical × sex → survival						-15.57	− 0.08	< 0.001	[-22.92, -8.23]
mental × sex → survival						-8.00	− 0.03	0.056	[-16.21, 0.21]
**Indirect effects**									
housework→ cognitive→ survival	1.23	-	0.642	[-3.95, 6.40]		6.73	-	0.425	[-9.79, 23.25]
house work → physical → survival	10.61	-	< 0.001	[4.32, 16.89]		11.29	-	0.009	[2.78, 19.80]
house work → mental → survival	2.56	-	0.040	[0.12, 5.00]		3.43	-	0.100	[-0.76, 7.61]
overall indirect effect	14.40	-	< 0.001	[5.90, 22.89]		21.45	-	0.027	[2.42, 40.48]
**Total effect (**housework → survival)	197.82	-	< 0.001	[161.41, 234.23]		275.95	-	< 0.001	[229.53, 322,37]

*Notes*. *b*: unstandardized path coefficient; *β*: standardized path coefficient; 95% *CI*: 95% confidence interval

To verify the reliability of these mediation effects, we performed several supplementary analyses, including a mediation model using the weighted housework score as the predictor variable, a reverse model examining whether housework mediated the relationship between health measures and survival, and analyses of time-lagged associations among housework, health measures, and survival. Results of these analyses are reported in the Supplementary Materials. Overall, the supplementary analyses supported the mediating effects of the health measures on the relationship between housework and survival.

Given that sex moderated the association between housework and number of days survived in the linear regression model, we tested a moderated mediation model in which sex moderated the multiple parallel mediation paths described above, with demographic covariates controlled for (see Model with Sex Moderation in Table 5). We found a significant moderating effect of sex on the relationship between the mediators and survival (sex × cognitive functioning → survival: unstandardized *b* = -210.19, *SE* = 18.03, *p* < .001, 95% *CI* = [-245.53, -174.85]; sex × physical health → survival: unstandardized *b* = -15.57, *SE* = 3.75, *p* < .001, 95% *CI* = [-22.92, -8.23]; sex × mental health → survival: unstandardized *b* = -8.00, *SE* = 4.19, *p* = .056, 95% *CI* = [-16.21, 0.21]), but not for the other relationships (*p* values > 0.1). Together with the linear regression and Cox proportional hazard results, these mediation results suggest that the weaker main effect of housework on survival among women (versus men) may be explained by the weaker associations between cognitive/physical/mental health and survival among women (versus men). Additional analyses after stratifying by sex revealed stronger mediation effects among men than among women (see Table 5). To be specific, the overall mediation effect (i.e., indirect effect) was significant among men (unstandardized *b* = 28.14, *SE* = 7.30, *p* < .001, 95% *CI* = [13.84, 42.45]) but not among women (unstandardized *b* = 15.24, *SE* = 17.58, *p* = .386, 95% *CI* = [-19.22, 49.70]). As for individual mediation effects, the mediating effects of physical health (*p* = .066) and mental health (*p* = .076) were marginally significant among women, whereas the mediating effects of cognitive functioning (*p* = .041) and physical health (*p* = .002) were significant among men.

**Table 5:** Path Coefficients for the Mediation Models for Men (N = 2,000) and Women (N = 2,000)

	Women’s Model					Men’s Model			
	*b*	*β*	*p*	95% *CI*		*b*	*β*	*p*	95% *CI*
**Regressions**									
housework→ survival	226.63	0.11	< 0.001	[137.03, 316.23]		236.46	0.18	< 0.001	[188.28, 284.65]
housework → cognitive	-0.03	− 0.01	0.688	[-0.16, 0.11]		0.06	0.04	0.033	[0, 0.12]
housework → physical	0.58	0.05	0.046	[0.01, 1.15]		0.55	0.07	0.001	[0.21, 0.88]
housework → mental	0.48	0.04	0.065	[-0.03, 0.98]		0.30	0.04	0.050	[0, 0.6]
cognitive → survival	223.44	0.37	< 0.001	[194.86, 252.02]		129.72	0.13	< 0.001	[93.67, 165.76]
physical → survival	16.58	0.10	< 0.001	[9.75, 23.4]		34.00	0.19	< 0.001	[27.9, 40.11]
mental → survival	24.88	0.14	< 0.001	[17.39, 32.36]		5.21	0.03	0.137	[-1.65, 12.08]
*(Regressions on covariates)*									
age → survival	-17.11	− 0.07	0.008	[-29.68, -4.53]		-123.20	− 0.46	< 0.001	[-132.99, -113.42]
education → survival	15.15	0.02	0.562	[-36.06, 66.35]		50.30	0.06	0.001	[20.33, 80.26]
marital → survival	485.99	0.17	< 0.001	[337.5, 634.47]		581.47	0.14	< 0.001	[409.1, 753.84]
living status → survival	290.71	0.08	0.001	[124.07, 457.36]		85.29	0.02	0.443	[-132.55, 303.13]
social status → survival	53.71	0.08	< 0.001	[23.7, 83.71]		-20.29	− 0.03	0.115	[-45.54, 4.96]
age → cognitive	-0.09	− 0.21	< 0.001	[-0.11, -0.07]		-0.06	− 0.22	< 0.001	[-0.07, -0.05]
education → cognitive	0.57	0.34	< 0.001	[0.5, 0.64]		0.24	0.30	< 0.001	[0.2, 0.27]
marital → cognitive	0.35	0.08	0.002	[0.13, 0.58]		-0.05	− 0.01	0.650	[-0.26, 0.16]
living status → cognitive	-0.10	− 0.02	0.467	[-0.36, 0.17]		0.17	0.03	0.198	[-0.09, 0.44]
social status → cognitive	-0.16	− 0.14	< 0.001	[-0.21, -0.12]		-0.02	− 0.03	0.223	[-0.05, 0.01]
age → physical	-0.07	− 0.04	0.092	[-0.15, 0.01]		-0.10	− 0.07	0.004	[-0.17, -0.03]
education → physical	0.42	0.07	0.004	[0.14, 0.7]		0.09	0.02	0.402	[-0.11, 0.29]
marital → physical	-0.56	− 0.03	0.243	[-1.51, 0.38]		-0.20	− 0.01	0.754	[-1.46, 1.06]
living status → physical	-0.78	− 0.04	0.170	[-1.89, 0.33]		0.90	0.03	0.261	[-0.67, 2.48]
social status → physical	0.25	0.06	0.009	[0.06, 0.44]		0.55	0.14	< 0.001	[0.38, 0.73]
age → mental	0.10	0.07	0.004	[0.03, 0.17]		0.07	0.06	0.016	[0.01, 0.14]
education → mental	0.17	0.03	0.176	[-0.08, 0.43]		0.28	0.07	0.002	[0.1, 0.46]
marital → mental	0.17	0.01	0.687	[-0.67, 1.02]		0.22	0.01	0.708	[-0.91, 1.34]
living status → mental	0.92	0.05	0.070	[-0.07, 1.9]		0.93	0.04	0.197	[-0.48, 2.35]
social status → mental	0.33	0.09	< 0.001	[0.16, 0.49]		0.24	0.07	0.003	[0.08, 0.4]
**Indirect effects**									
housework → cognitive→ survival	-6.19	-	0.688	[-19.22, 49.70]		7.96	-	0.041	[0.31, 15.60]
housework → physical → survival	9.58	-	0.066	[-0.63, 19.79]		18.62	-	0.002	[6.75, 30.49]
housework → mental → survival	11.85	-	0.076	[-1.25, 24.94]		1.56	-	0.236	[-1.02, 4.15]
overall indirect effect	15.24	-	0.386	[-19.22, 49.70]		28.14	-	< 0.001	[13.84, 42.45]
**Total effect** (housework → survival)	241.87	-	< 0.001	[146.17, 337.57]		264.61	-	< 0.001	[214.72, 314.49]

*Notes*. *b*: unstandardized path coefficient; *β*: standardized path coefficient; 95% *CI*: 95% confidence interval

## Discussion

The aim of the present study was to examine the longitudinal association between housework (at the baseline) and subsequent survival days over a 14-year span among adults aged 65 years and above in Hong Kong, and to investigate the potential mediating roles of physical health, cognitive functioning, and mental health (at the baseline) in this association. The results of a parallel mediation model showed a significant association between higher engagement in housework and reduced mortality, and this link was partially mediated by better physical health and mental health, regardless of whether controlling for demographic covariates (i.e., age, sex, education, marital status, living alone, and subjective social status). No evidence was found for a mediating role of cognitive functioning in the housework-survival association.

As hypothesized, older adults who engaged in more types of housework at the baseline tended to survive longer (higher number of days survived post-baseline). This finding aligns with previous research linking housework with reduced all-cause mortality in old age [3, 12]. Housework can be physically strenuous and cognitively and emotionally demanding, suggesting multiple mechanisms by which engaging in housework may affect health and mortality. The present study extends previous work by distinguishing between three potential mechanisms underlying the housework-survival relationship (i.e., physical health, cognitive functioning, and mental health).

Physical health (self-reported physical symptoms) partially mediated the association between housework and survival time, as hypothesized. This mediation effect may be attributed to the well-established physical health benefits of engaging in physical activity [37]. Compared to work-related and leisure activities, housework activities make the largest contribution to total physical activity hours among older adults, particularly among older women [12], and mortality reduction has been linked specifically with household-related physical activity [12], or non-leisure-time physical activity [3]. Spending more time on housework has been found to be associated with a higher likelihood of meeting standard physical activity guidelines [2] and better self-reported health among older adults [1]. This is consistent with the possibility that the health benefits of physical activity partially explain the positive link between housework and survival.

Mental health was also found to partially mediate the relationship between housework and survival. As mentioned above, engaging in housework may increase individuals’ levels of physical activity, and physical activity in turn has been found to have a protective effect on mental health. A recent review synthesizing studies on older adults in South and Southeast Asia has found that greater engagement in physical activities is associated with lower depression risk and better sleep quality [38]. Research during the COVID-19 pandemic has also affirmed the protective effects of housework in reducing depression risk [39]. A likely explanation is that physical activity could be a long-term lifestyle factor that promotes well-being and health in later life [40]. However, when compared with activities outside the home, such as volunteering work, Fekete et al., (2018) found that housework showed a weaker association with better mental health among older adults in Switzerland [41]. This discrepancy could be related to cultural differences, such that older participants in East Asia may find doing housework fulfills their familial roles and self-values, while European older adults prefer volunteering work to enhance their social connections and sense of belonging.

Counter to our hypothesis, a mediating role of cognitive functioning on housework-survival associations was not found. One possible explanation is that the types of housework examined might have become habitual and less cognitively stimulating when repeated for years. In line with this speculation, in an analysis of data from the Chinese Longitudinal Healthy Longevity Survey (CLHLS), Zhang et al., (2020) found that the cognitive benefits of engaging in housework and farming were much lower than those of engaging in comprehensive tasks [20]. Moreover, although engaging in everyday activities could lead to reduced risk of Alzheimer’s disease and dementia, these activities usually involved information processing [42]. Therefore, it seems that the nature of housework should be considered when understanding its effects on cognitive functioning, and housework involving information processing and comprehensive tasks, rather than redundant, customary labor, may bring more cognitive stimulation.

Sex moderated the overall housework-survival association, suggesting that the effect of doing housework on survival may be stronger among men than among women. Specifically, the sex-stratified mediation analyses revealed that older women’s housework engagement was not significantly or marginally significantly associated with improvements in health measures, although improvements in these health measures were significantly associated with increases in women’s survival days. This finding is consistent with some previous research which has linked housework with reduced mortality risk for older men, but not older women [3, 12]. Greater housework hours or domestic workload has also been linked with worse self-reported physical health for women but not for men in studies of working-age adults [8, 9] and retired adults [11]. Moreover, according to the moderated mediation model, the weaker indirect effect among women may be attributed to the weaker links between physical/mental/cognitive health measures and survival among women. This finding may be related to women’s greater longevity compared to men in general, despite suffering from more chronic conditions and depressive symptoms [e.g., 43, 44], and suggests that lifestyle factors may be important in understanding sex differences in mortality. Moreover, since women and men usually engage in different types of housework, future studies should investigate the effects of independent components of housework engagement on health among older men and women.

The present study has the strength of using a 14-year longitudinal dataset, enabling us to examine time-ordered associations between housework and mortality spanning a substantial period of older adulthood. However, there are also several limitations of this study. First, engagement in housework was captured using subjective measures, which are prone to reporting biases. Future studies may consider measuring housework engagement using intensive longitudinal measures (to reduce reliance on memory) or objective measures (e.g., activity sensors or monitoring system). Second, this is a correlational study and reversed models suggest that the relationships between housework and physical health and cognitive functioning may be bi-directional (see Supplementary Materials). For example, reduced engagement in housework may be a warning sign for physical decline or cognitive decline, as a healthy body and mind may enable individuals to do more housework, rather than the other way around. Moreover, although the current study includes cross-lagged analyses (see the Supplementary Materials), it only includes two waves of measurement of housework and health-related variables. Future work with more complete measurements of the key variables and more assessment waves may help to better examine dynamic relationships among these variables over time.

Third, many risk factors (e.g., lifestyle factors, genetic factors) could contribute to health and mortality but were not controlled for in the current study. For instance, participants less engaged in housework were less likely to live actively in general. It might be the case that weaker engagement in activities other than housework undermined these participants’ health and survival, which might confound our current findings. Future studies could consider controlling for these variables to account for their potential confounding effects when examining the effects of housework on mortality. A fourth potential limitation of the current study is the generalizability of the findings. In contrast to the majority of previous research on housework, health, and survival conducted with European and American samples, the present study targeted East Asian older adults, an understudied population. The meaning and impact of housework activities may differ across cultures. For example, among East Asian older adults, housework activities may be shaped by collectivistic values emphasizing familial role fulfillment, potentially resulting in greater health and well-being benefits from engaging in housework. Further studies are needed to examine whether cultural factors may moderate our findings.

## Conclusions

The present study, though correlational, suggests that engaging in housework in older age is associated with better physical health and mental health, which in turn contribute to prolonged survival. The findings advance our understanding of the impact of housework on mortality and provide critical insights for tailoring housework-based health promotion programs for older adults. Practical uses of these findings may include positive messaging encouraging both older men and women to engage in housework for health and longevity benefits (in spite of gender stereotypes), as well as emphasizing how housework can be seen less as a chore and more as a form of physical exercise. Future work may consider developing at-home or housework-based interventions to enhance physical and mental health, particularly among home-bound older adults.

## Electronic supplementary material

Below is the link to the electronic supplementary material.

Supplementary Material 1

## Data Availability

The data that support the findings of this study are available from Mr. & Ms. Os Hong Kong cohort study (http://www.jococ.org/en/mros-msos.php), but restrictions apply to the availability of these data, which were used under license for the current study, and so the data are not publicly available. Data are, however, available from Prof. Timothy Kwok (tkwok@cuhk.edu.hk) upon reasonable request and with permission of the Jockey Club Centre for Osteoporosis Care and Control.
