# Supplementary material for: The perks of doing housework: Longitudinal associations with survival and underlying mechanisms
Source: BMC Geriatr. 2023 Jun 6;23:355. doi: 10.1186/s12877-023-04039-1 (PMC10242587; doi:10.1186/s12877-023-04039-1)
Supplement: Supplementary file 1 — Supplementary Material 1 [file 12877_2023_4039_MOESM1_ESM.pdf]

Supplementary Materials

In the main text, we present the results of a parallel mediation analysis (see Table 4 and Figure 1 in the main text), which show significant mediating effects of health measures (specifically, physical health and mental health) on the relationship between housework and survival. To verify the reliability of the mediation effects, we conducted several supplementary analyses, presented below.

First, we performed a multiple parallel mediation model using the weighted housework score, in which the original housework activity engagement score was multiplied by the physical activity weights as suggested by PASE [27] (see Table S1 below). The results showed a significant mediating effect of physical health (unstandardized $b = 0.30$, $p = .001$) but not of cognitive functioning or mental health (p values > .10), after controlling for demographic covariates. This result suggests a robust mediating effect of physical health on the homework-survival association, regardless of the way that housework is calculated.

Second, as both housework and the mediator variables reported in the mediation analysis above were measured at the baseline, one may argue for an alternative interpretation, that is, it might be the case that housework engagement mediated the relationship between the health measures (i.e., cognitive functioning, physical health, and mental health) and survival. To test for this possibility, a reverse mediation model (i.e., using housework as a mediator of the association between the health measures and survival) was tested (see Supplementary Table S2 below). The results showed significant mediating effects of housework on the relationship between physical health/mental health and survival days (p values < .01). This result does not rule out the alternative possibility that housework, rather than the health measures, played a mediating role. That is, older adults with better health status might be more engaged in housework, which may in turn prolong their survival.

Third, to help rule out the above alternative explanation and further confirm the mediating roles of the health measures, we performed a cross-lagged mediation analysis (see Supplementary Figure S1 and Table S3 below). Specifically, we examined 1) whether the health measures at the year-7 follow-up mediated the relationship between baseline housework and survival days by the year-14 follow-up, and 2) whether housework at the year-7 follow-up mediated the relationship between the baseline health measures and survival days by the year-14 follow-up. Participants who were deceased by the year-7 follow-up had no scores on the

housework and health measures at this measurement point. We performed multiple imputation for these missing data using the *mice* package [33] in R [34]. The direct effects and mediation effects (i.e., indirect effects) of interest are listed in Supplementary Table S3 below. The results showed significant mediating effects of year-7 health measures (specifically, physical and mental health) on the relationship between baseline housework and year-14 survival, but non-significant mediating effects of year-7 housework on the relationships between baseline health measures and year-14 survival. This cross-lagged mediation analysis provides evidence for the mediating roles of physical and mental health in the relationship between housework and survival days, as reported in the main text.

However, as noted above, some participants had already passed away by the year-7 follow-up, and their health measures were therefore not available. From this perspective, each model in the present study has different advantages and drawbacks. While the primary analyses fall short in accounting for the temporal or “causal” association between housework engagement and health outcomes, the cross-lagged model involved imputation of follow-up data on mediators for participants who passed away before the 7-year follow-up. We have therefore chosen to treat the time-lagged mediation analysis as a supplementary analysis, and we draw our conclusions for this study based on the original mediation analysis reported in the main text (i.e., baseline housework → baseline health measures → number of days survived by the year-14 follow-up). In short, the results of these supplementary analyses support the main findings of the mediation analysis reported in the main text, that is, health measures (specifically, physical health and mental health) mediated the relationship between housework and survival.

Supplementary Table S1

Path Coefficients for the Mediation Model (Weighted Housework as the Predictor Variable)

Path	Mediation Model (Housework being Weighted)				Mediation Model Without Covariates (Housework being Weighted)			
	b	*β*	*p*	95% CI	*b*	*β*	*p*	95% CI
**Regressions**								
housework_w→ survival	4.63	.08	< .001	[2.88, 6.38]	8.17	.14	< .001	[6.36, 9.98]
housework_w → cognitive	0.001	.01	.385	[-0.001, 0.003]	0.004	.05	.004	[0, 0.01]
housework_w → physical	0.02	.06	< .001	[0.01, 0.03]	0.02	.06	< .001	[0.01, 0.03]
housework_w → mental	0.01	.03	.089	[-0.001, 0.02]	0.003	.01	.454	[-0.01, 0.01]
cognitive → survival	44.62	.06	< .001	[19.52, 69.72]	72.09	.10	< .001	[49.31, 94.88]
physical → survival	14.85	.08	< .001	[9.47, 20.22]	9.61	.05	.001	[4.11, 15.11]
mental→ survival	3.87	.02	.208	[-2.16, 9.9]	0.38	.00	.906	[-5.96, 6.73]
[Regressions on Covariates]								
age → survival	-79.41	-.27	< .001	[-88.67, -70.16]				
sex→ survival	581.19	.19	< .001	[477.74, 684.64]				
education → survival	36.82	.04	.018	[6.31, 67.34]				
marital → survival	139.29	.04	.031	[12.36, 266.22]				
living status → survival	68.24	.02	.381	[-84.56, 221.04]				
social status → survival	-3.85	-.01	.740	[-26.62, 18.92]				
age → cognitive	-0.08	-.19	< .001	[-0.001, 0.003]				
sex→ cognitive	-0.91	-.22	< .001	[-0.09, -0.06]				
education → cognitive	0.38	.30	< .001	[-1.03, -0.79]				
marital → cognitive	0.32	.07	< .001	[0.34, 0.41]				
living status → cognitive	-0.05	-.01	.623	[0.17, 0.48]				
social status → cognitive	-0.10	-.10	< .001	[-0.24, 0.14]				
age → physical	-0.08	-.05	.003	[-0.13, -0.03]				
sex→ physical	-4.08	-.24	< .001	[-4.65, -3.51]				
education → physical	0.24	.05	.005	[0.07, 0.41]				
marital status → physical	-0.40	-.02	.287	[-1.13, 0.33]				

living status → physical	-0.40	-.02	.377	[-1.28, 0.48]				
social status → physical	0.39	.09	< .001	[0.26, 0.52]				
age → mental	0.07	.05	.002	[0.03, 0.12]				
sex→ mental	-0.60	-.04	.020	[-1.1, -0.09]				
education → mental	0.20	.04	.008	[0.05, 0.35]				
marital status → mental	0.14	.01	.684	[-0.52, 0.79]				
living status → mental	0.77	.04	.054	[-0.01, 1.56]				
social status → mental	0.29	.08	< .001	[0.17, 0.41]				
**Indirect Effects**								
housework_w → cognitive → survival	0.04	-	.400	[-0.05, 0.14]	0.26	-	.008	[0.07, 0.46]
housework_w → physical → survival	0.30	-	.001	[0.11, 0.48]	0.18	-	.013	[0.04, 0.32]
housework_w → mental → survival	0.03	-	.312	[-0.03, 0.09]	0.001	-	.907	[-0.02, 0.02]
**Total Effect**								
housework_w → survival	5.00	-	< .001	[3.25, 6.75]	8.62	-	< .001	[6.80, 10.43]

Notes. In this model, housework was calculated by taking the sum of all six activity engagement items (0 = no engagement, 1 = engaged) after multiplying them by the physical activity weights (*M* = 43.7, *SD*= 25.62, range [0, 171]) suggested in Washburn et al., 1993. Housework w. weighted housework; *b*. unstandardized coefficient; *β*. standardized coefficient; 95% *CI*. 95% confidence interval.

Supplementary Table S2

Path Coefficients for the Reversed Mediation Model (Housework as the Mediator)

Path	Reversed Mediation Model				Reversed Mediation Model Without Covariates			
	b	β	*p*	95% *CI*	*b*	β	*p*	95% *CI*
**Regressions**								
housework → survival	183.44	.13	< .001	[147.87, 219.01]	519.36	.15	< .001	[396.87, 641.84]
cognitive → housework	<.001	<.001	.980	[-0.01, 0.02]	0.005	.01	.467	[-0.01, 0.02]
physical → housework	0.01	.06	.001	[0.002, 0.01]	0.004	.04	.014	[0.001, 0.01]
mental → housework	0.005	.04	.012	[0.001, 0.01]	0.002	.02	.191	[-0.001, 0.01]
cognitive → survival	80.65	.14	< .001	[63.53, 97.77]	160.37	.11	< .001	[109.23, 211.51]
physical → survival	19.94	.14	< .001	[16.24, 23.64]	11.87	.03	.064	[-0.69, 24.43]
mental → survival	7.45	.05	< .001	[3.31, 11.59]	3.30	.01	.651	[-10.99, 17.6]
[Regressions on covariates]								
age → housework	-0.03	-.16	< .001	[-0.03, -0.02]				
sex → housework	0.14	.08	< .001	[0.08, 0.2]				
education → housework	-0.02	-.03	.084	[-0.03, 0]				
marital → housework	0.11	.06	.005	[0.03, 0.18]				
living status → housework	-0.28	-.11	< .001	[-0.37, -0.19]				
social status → housework	-0.02	-.05	.002	[-0.04, -0.01]				
age → survival	-104.57	-.45	< .001	[-110.85, -98.28]				
sex → survival	978.75	.41	< .001	[904.85, 1052.7]				
education → survival	53.99	.07	< .001	[32.5, 75.49]				
marital → survival	233.97	.09	< .001	[145.55, 322.38]				
living status → survival	117.76	.03	.028	[12.91, 222.62]				
social status → survival	3.20	.01	.691	[-12.55, 18.95]				
**Indirect effects**								
cognitive → housework	0.03	-	.980	[-2.72, 2.79]	2.57	-	< .001	[-4.37, 9.50]

→ survival								
physical→ housework → survival	1.04	-	.001	[0.42, 1.66]	2.08	-	.019	[0.34, 3.81]
mental → housework → survival	0.85	-	.014	[0.17, 1.53]	1.27	-	.196	[-0.65, 3.18]
**Total effects**								
cognitive → survival	80.68	-	< .001	[63.35, 98.02]	162.94	-	< .001	[111.32, 214.55]
physical → survival	20.98	-	< .001	[17.24, 24.72]	13.95	-	.031	[1.29, 26.60]
mental → survival	8.30	-	< .001	[4.11, 12.49]	4.57	-	.621	[-9.85, 18.99]

Notes. *b*. unstandardized coefficient; *β*. standardized coefficient; 95% *CI*. 95% confidence interval.

Supplementary Table S3

Path Coefficients for the Cross-Lagged Mediation Model

Path	*b*	*β*	*p*	*95% CI*
Baseline → Yr7				
housework_baseline → housework_yr7	0.0001	.0001	.992	[-0.02, 0.02]
housework_baseline → cognitive_yr7	0.14	.07	< .001	[0.08, 0.20]
housework_baseline → physical_yr7	0.76	.08	< .001	[0.45, 1.07]
housework_baseline → mental_yr7	0.33	.03	.036	[0.02, 0.63]
cognitive_baseline → cognitive_yr7	0.01	.01	.526	[-0.02, 0.04]
cognitive_baseline → housework_yr7	-0.03	-.07	< .001	[-0.04, -0.02]
physical_baseline → physical _yr7	0.14	.13	< .001	[0.11, 0.17]
physical_baseline → housework_yr7	0.02	.18	<. 001	[0.015, 0.021]
mental_baseline → mental _yr7	0.10	.09	< .001	[0.07, 0.14]
mental_baseline → housework_yr7	0.01	.11	<. 001	[0.01, 0.02]
Yr7 → Yr14				
housework_yr7 → survival_yr14	0.37	.0003	.989	[-51.68, 52.42]
cognitive_yr7 → survival_yr14	-12.42	-.02	.199	[-31.38, 6.54]
physical_yr7 → survival_yr14	-5.29	-.05	<. 001	[-7.78, -2.81]
mental_yr7 → survival_yr14	-9.17	-.09	<. 001	[-11.67, -6.67]
Baseline → Yr14				
housework_baseline → survival_yr14	-0.24	-.0002	.989	[-34.38, 33.90]
cognitive_baseline → survival_yr14	-10.46	-.02	.224	[-27.29, 6.38]
physical_baseline → survival_yr14	-14.62	-.13	<. 001	[-18.31, -10.93]
mental_baseline → survival_yr14	-18.71	-.149	<. 001	[-22.95, -14.48]
Indirect effects of housework on survival				
housework_baseline → cognitive_yr7 → survival_yr14	-1.69	-	.218	[-4.38, 1.00]
housework_baseline → physical_yr7 → survival_yr14	-4.01	-	.002	[-6.50, -1.52]
housework_baseline → mental_yr7 → survival_yr14	-3.00	-	.044	[-5.92, -0.08]

Indirect effects of health measures on survival				
cognitive_baseline → housework_yr7 → survival_yr14	-0.01	-	.989	[63.35, 98.02]
physical_baseline → housework_yr7 → survival_yr14	0.007	-	.989	[-0.95, 0.96]
mental_baseline → housework_yr7 → survival_yr14	0.004	-	.989	[-1.55, 1.53]

Note. Demographic covariates were controlled for paths leading to the mediator variables and the outcome variable, but the estimates of these covariates are not displayed in this table. *b*. unstandardized coefficient; *β*. standardized coefficient; 95% *CI*. 95% confidence interval.

Supplementary Figure S1

Visual Representation of the Cross-Lagged Mediation Model

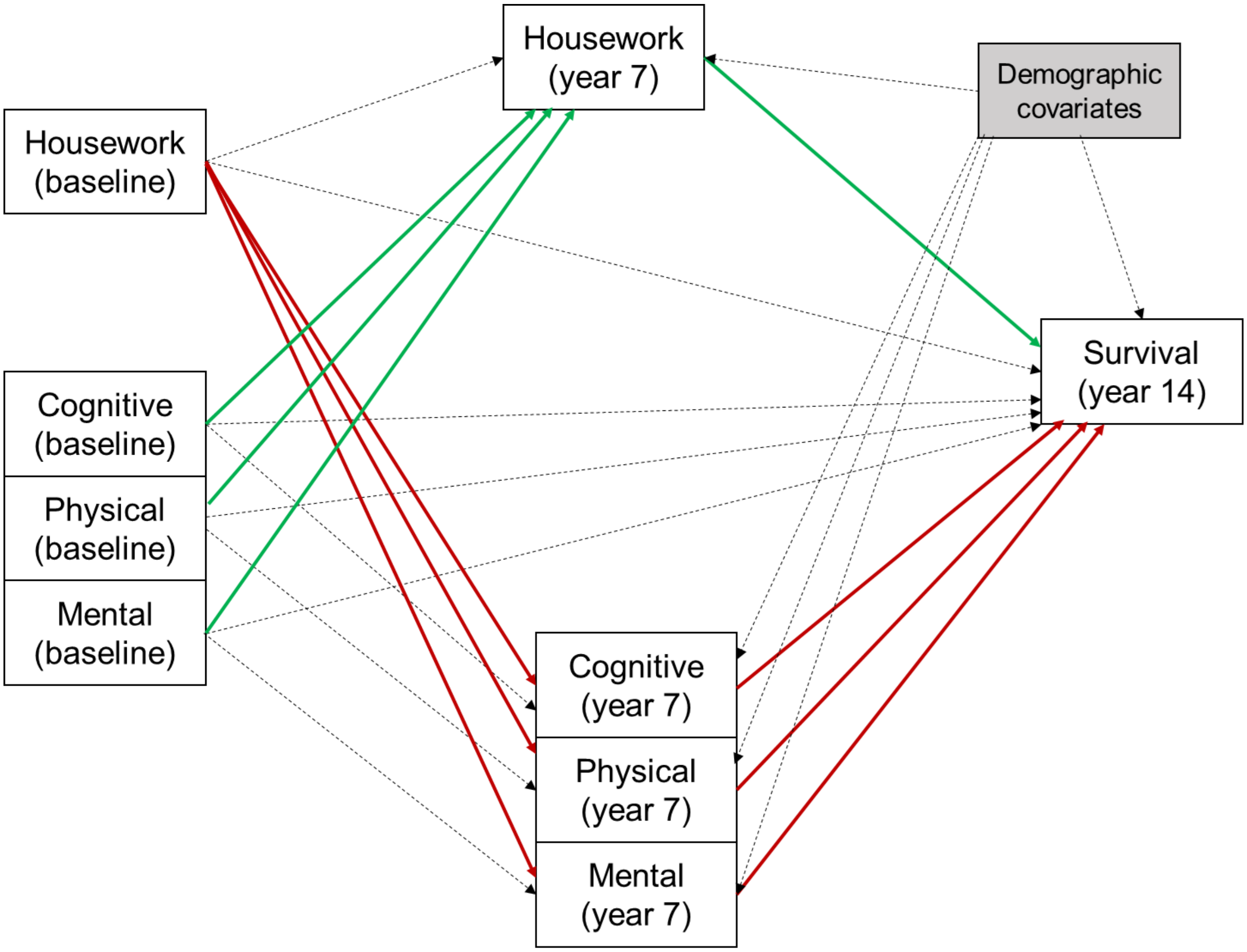

Note. A cross-lagged mediation model. The red lines indicate the indirect effects of baseline housework on year-14 survival via year-7 health measures (cognitive functioning, physical health, and mental health). The green lines indicate the indirect effects of baseline health measures on year-14 survival via year-7 housework. Demographic covariates (age, sex, education, marital status, living alone, and subjective social status) were controlled for the paths leading to both the mediation and the outcome variables. For detailed results, see Supplementary Table S3.
